# Supplementary material for: Rapid threat assessment in the Drosophila thermosensory system
Source: Nat Commun. 2023 Nov 3;14:7067. doi: 10.1038/s41467-023-42864-5 (PMC10624821; doi:10.1038/s41467-023-42864-5)
Supplement: Supplementary file 1 — Supplementary Information [file 41467_2023_42864_MOESM1_ESM.pdf]

Supplementary material for:

Rapid threat assessment in the Drosophila thermosensory system

by

Jouandet et al.

(Corresponding author: marco.gallio@northwestern.edu)

Included:

Supplementary Figure 1, related to Figure 2

Supplementary Figure 2, related to Figure 4

Supplementary Figure 3, related to Figure 5

Supplementary Table 1, related to Figure 2

Supplementary Table 2, related to Figure 3

Supplementary Table 3, related to Figure 7

Supplementary Table 4, Table of Genotypes

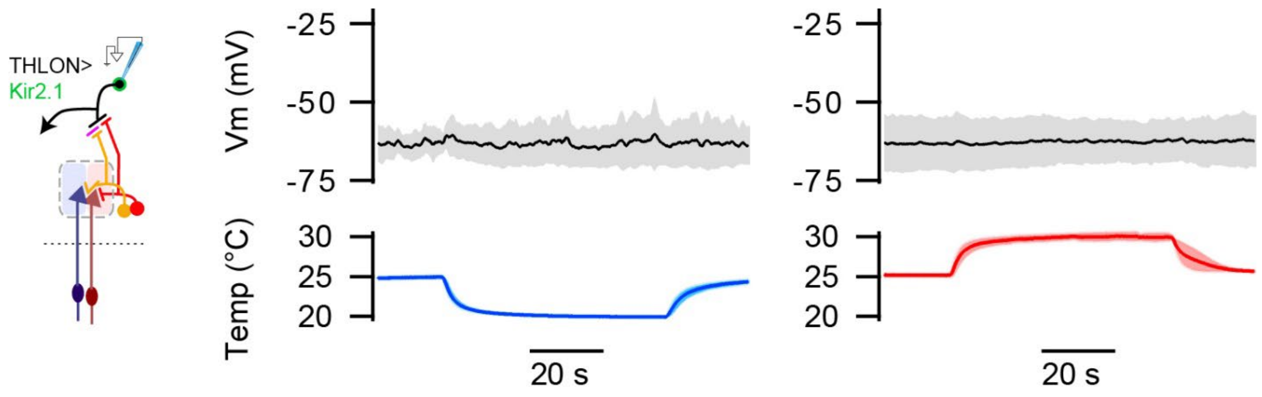

Supplementary Figure 1, related to Figure 2. Expression of Kir2.1 in TLHONs produces hyperpolarization and silences responses to thermal stimuli.

TLHONs were recorded in 2-photon guided patch-clamp in flies of the genotype: TLHON-Gal4, UAS-Kir2.1, UAS-CD8:GFP. (Left) schematic of recording configuration. (Right) traces acquired in whole cell patch clamp mode while challenging the preparation with cold (blue, left) or hot (red, right) step stimuli. (Top) average membrane potential; (Bottom) temperature recording (cold n = 4 cells, 2 animals; and hot n = 3 cells, 2 animals; bold line = mean; shading = +/- SEM).

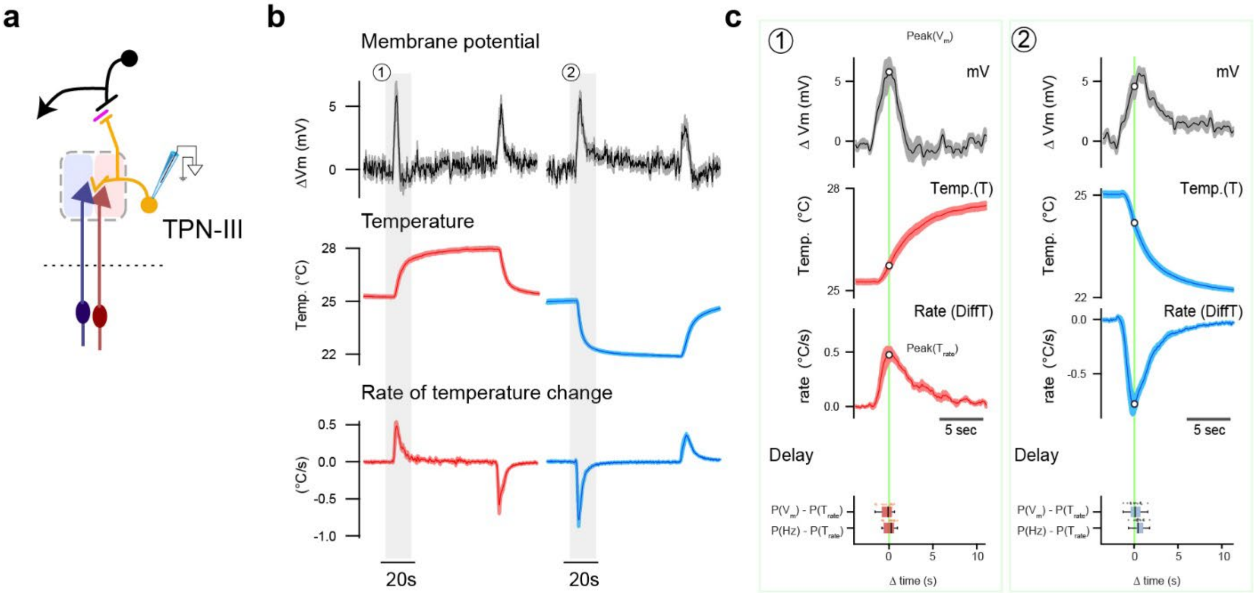

Supplementary Figure 2, related to Figure 4. TPN-III ON responses correlate with peak rate of temperature change.

TPN-III ON responses peak in correspondence with the fastest rate of heating or cooling. (**a**) Experiment schematic. (**b**) TPN-III filtered membrane potential responses (top traces), stimulus temperature (middle traces), and rate of thermal change (temporal derivative of temperature stimuli; bottom traces). (**c**) Green boxes are x-axis expansions of grey shaded regions in (**b**) as indicated. Delay (shown as boxplots) is quantified as the difference between the timing of the peak membrane potential or the peak firing rate and the timing of the peak rate of thermal change and shows temporal locking between peak response and peak stimulus rate (N=14 cells/7 animals, trace line and shading indicate mean ± SEM; boxplots: black line = median, box = interquartile range, whiskers = range, dots = individual cells).

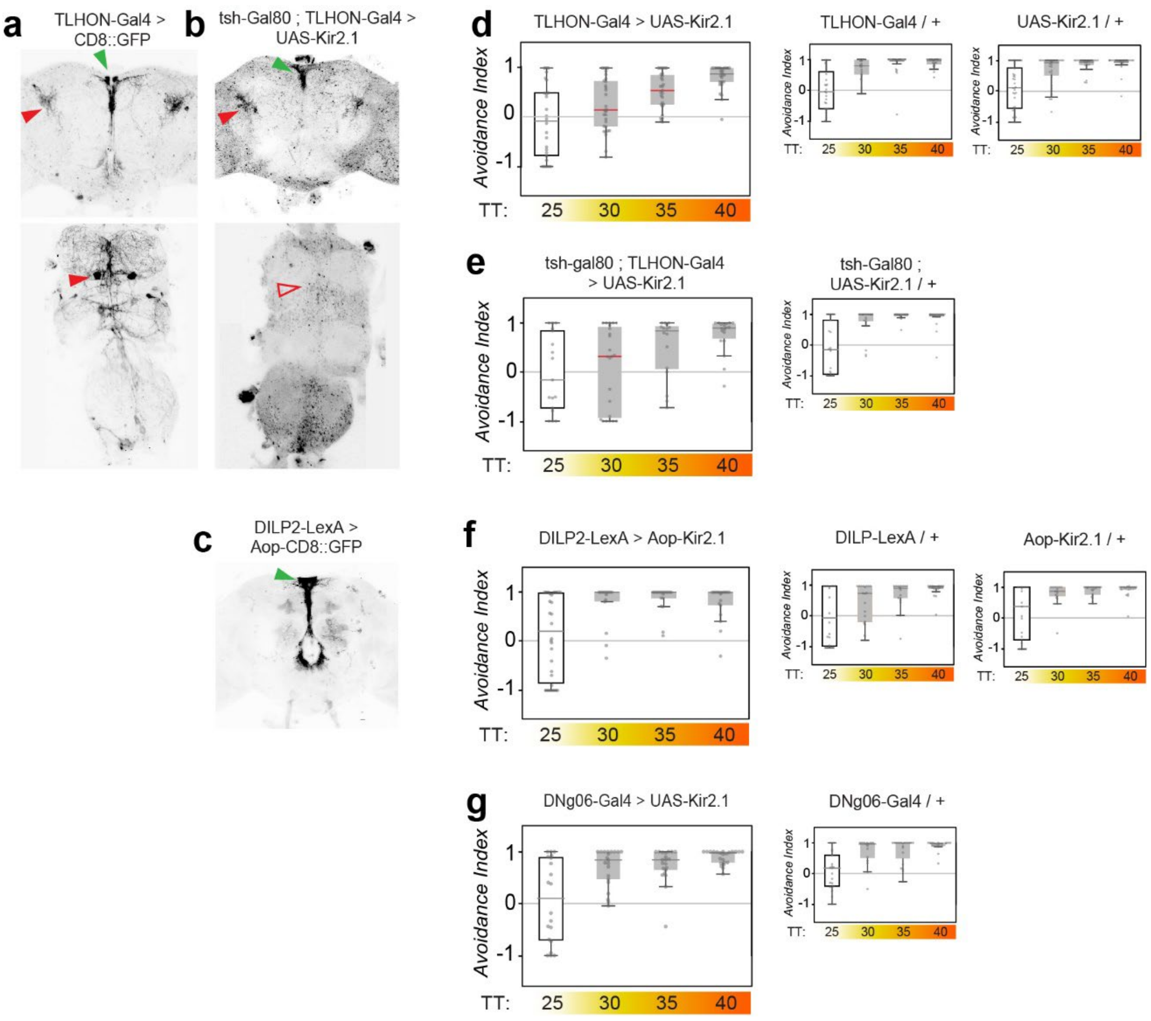

Supplementary Figure 3, related to Figure 5. TLHON-Gal4 full expression pattern and controls for off-target expression

(**a**) TLHON-Gal4 expression pattern in the brain and ventral nerve cord revealed by expression of CD8:GFP. Brain: Green arrowheads = Insulin-producing cells (IPCs). Red arrowheads = TLHONs. VNC: Red arrowheads = cell bodies. (**b**) tshirt-Gal80 (tsh-Gal80) effectively suppresses VNC expression (empty arrowhead). (**c**) The expression pattern of Dilp2-LexA > CD8:GFP is shown for reference. (**d-g**) Single fly temperature preference behavior suggests the phenotype of TLHON-Gal4 mediated silencing is not due to off-target expression in the VNC or DILPs. Single fly heat avoidance indexes for (**d**) TLHON-Gal4 > UAS-Kir2.1 and controls, (**e**) tshirt-gal80; TLHON-gal4/UAS-Kir2.1 and controls, (**f**) Dilp2-LexA > AoP-Kir2.1, and controls (see expression pattern of Dilp2-LexA in **d**), (**g**) DNg06-Gal4 > UAS-Kir2.1 and controls (note that, while TLHON-Gal4 mediated expression of GFP in DNg06 is at the detection limit, we tested this cell type out of an abundance of caution). In (**d-g**) gray dots each represent the avoidance index in 1 trial for 1 fly. In all boxplots, the edges of the boxes are the first and third quartiles, a solid line marks the median, and whiskers delimit the data range, a solid red median line denotes a significant interaction between experimental and control animals (2-way ANOVA, $p < 0.05$, N = 14-38, see Statistics table for precise N values for each test). TT = test temperature in °C; white to orange scale = 25 to 40°C.

		LHPV2a									
		**724820565**	**758903321**	**822005494**	**822009511**	**822684007**	**853717974**	**886130319**	**5813014218**	**5813041244**	**5813090530**
		LHPV2a postsynaptic									
TPN-III pre-	**819993149**	0	1	2	0	0	1	6	0	2	1
	**850703925**	1	13	7	0	1	18	4	12	8	7
	**850708902**	1	8	8	0	1	16	3	27	11	10
	**1037293275**	1	0	0	0	20	0	0	3	1	0
	**1630734360**	3	2	2	0	0	4	11	5	4	1
	**1733677514**	2	0	0	3	9	0	1	2	1	0
	**1943811736**	2	0	0	0	0	0	0	0	0	0
	**5813044177**	0	2	5	0	0	2	17	1	9	0
	**5813083723**	0	1	0	3	0	0	0	0	0	0
LHPV2g pre-	**882995659**	0	8	1	0	0	37	1	0	0	10
	**914027038**	0	7	5	0	0	26	3	3	0	6
		LHPV2a presynaptic									
TLHON post-	**882995659**	0	2	20	1	2	1	15	4	12	0
	**914027038**	0	4	11	0	1	1	11	7	6	0

		LHAV		LHPV	
		**574377845**	**1037510115**	**1006146837**	**913341138**
TPN pre-	TPN ID	postsynaptic			
	**1755556097**	13	0	4	0
	**1975878958**	124	23	1	10
	**2065197353**	0	0	18	4
	**2069644133**	0	0	10	4
	**5813040515**	16	0	7	0
		presynaptic			
TLHON post-	**882995659**	16	8	13	8
	**914027038**	14	6	3	4

		TLHON post-	
		**882995659**	**914027038**
TPN-V pre-	**1975878958**	26	30

Supplementary Table 1, related to Figure 2. Connectivity between TPN-IIIs, LHPV2as and TLHONs (LHPV2gs) and additional thermosensory drive to TLHONs. (See Figure 2 and methods for details). Synaptic connections between individual neurons for TPN-III thermosensory circuit with TLHONs: TPN-IIIs to LHPV2a, TLHONs to LHPV2a, LHPV2a to TLHONs. Synaptic connections for direct and indirect thermosensory pathways to TLHONs: TPNs to LHAV/LHPV, LHAV/LHPV to TLHONs, TPN-V to TLHONs.

			TLHON post-	
			882995659	914027038
OPN pre-	VL2p_adPN	**1944507292**	27	30
	VL2a adPN	**5813069089**	24	4
	VM4 lvPN	**1850024931**	10	12
	VM4 lvPN	**726207450**	6	6
LHPV pre-	LHPV4i1	**699515201**	8	12
	LHPV4a6	**729867427**	13	4
	LHPV6k1	**758239379**	6	4
	LHPV2a3	**759582415**	14	6
	LHPV4j1_a	**759888294**	5	6
	LHPV4i1	**759888465**	5	6
	LHPV4j1_b	**760570054**	7	5
	LHPV2a3	**789934899**	10	5
	LHPV2a1_d	**822005494**	20	11
	LHPV4k1_a	**851314221**	11	3
	LHPV2a1_c	**886130319**	15	11
	LHPV4g1	**913341138**	8	4
	LHPV4f1_a	**942677658**	10	5
	LHPV1d1	**1006146837**	13	3
	LHPV2a1_c	**5813014218**	4	7
	LHPV2a1_c	**5813041244**	12	6
	LHPV6g1	**5813049920**	18	7
LHAV pre-	LHAV3f1	**574377845**	16	14
	LHAV4a2	**637121971**	5	5
	LHAV1a3	**823999645**	27	12
	LHAV2b2_a	**851961337**	22	9
	LHAV2b2_a	**852302504**	16	8
	LHAV1a3	**855439675**	18	4
	LHAV2b2_b	**1037510115**	8	6
	LHAV4f1	**1259528984**	11	8

		**LHPV/LHAV post-**
**OPN Class pre-**	VA1v_vPN	393
	VL2p_adPN	349
	DA1_lPN	190
	DA4l_adPN	183
	DP1m_adPN	148
	VC5_adPN	145
	DC2_adPN	132
	VL2a_adPN	118
	VC3m_lvPN	98
	VM1_lPN	95
	DA4m_adPN	85
	VA6_adPN	84
	VL1_vPN	77
	VM7d_adPN	75
	VL2a_vPN	70
	VA1v_adPN	63
	VM4_lvPN	59
	VA7l_adPN	52
	DP1l_adPN	44
	DA2_lPN	40
	VC1_lPN	37
	DC1_adPN	33
	DL1_adPN	28
	VL1_ilPN	27
	DC3_adPN	25
	DC4_adPN	23
	DL2d_adPN	23
	VL2p_vPN	20
	VA2_adPN	19
	VM7v_adPN	17
	DL4_adPN	16
	V_l2PN	14
	VC5_lvPN	14
	VC4_adPN	10

Supplementary Table 2, related to Figure 3. Olfactory drive to TLHONs. (See Figure 3 and methods for details). Synaptic connections between individual neurons for direct and indirect olfactory PNs: OPNs to TLHONs, OPNs to LHAV/LHPV, LHAV/LHPV to TLHONs. (Right) Combined synaptic connections for identified OPN glomerulus to LHAV/LHPV.

		LH/VLP post-						
		**792326206**	**821612285**	**851961337**	**852302504**	**915105429**	**1037510115**	**5813021533**
TLHON pre-	882995659	31	22	20	18	39	39	51
	914027038	18	20	23	21	32	52	39

		LHAD1
		**792326206**
LH/VLP pre-	**821612285**	50
	**851961337**	40
	**852302504**	44
	**1037510115**	22
	**5813021533**	66

		DN post										
		**887195902**	**1226887763**	**1281324958**	**1405231475**	**1406966879**	**1466998977**	**1565846637**	**2307027729**	**5813023322**	**5813024015**	**5813050455**
LH/VLP pre-	**792326206**	0	8	25	27	0	0	15	201	336	24	3
	**915105429**	0	41	5	6	18	16	1	0	145	27	0
	**5813021533**	29	0	0	0	0	0	0	0	3	0	22

Supplementary Table 3, related to Figure 7. Connectivity from TLHONs to LHAD1, LHAV2b, PVLP076, AVLP053 and to downstream DNs. (See Figure 7 and methods for details)**.** Synaptic connections between individual neurons downstream of TLHONs: TLHONs to LH/VLP, LH/VLP feedback to LHAD1, LH/VLP to DNs

Figure	Genotype	Panel
1	VT040053-GAL4 > UAS.CD8::GFP	b
	R22C06-GAL4 > UAS.CD8::GFP	b
	VT040053-GAL4.DBD; R22C06 AD> UAS.CD8::GFP	b,g,h,j-l
	VT040053-GAL4.DBD; R22C06 AD> UAS.Kir2.1	d
	UAS.Kir2.1/ +	e
	VT040053-GAL4.DBD; R22C06 AD/ +	e
	GR28B.d-LexA> LexAop.CsChrimson, VT040053-GAL4.DBD; R22C06 AD> UAS.CD8::GFP	g,h
	R77C10-GAL4 > UAS.CsChrimson, R22C06-LexA>LexAop::GFP	g,h
2	MB247dsRed, R22C06-LexA > LexAop::GFP, nSyb-GAL4 > UAS.C3PA	a,b
	VT061933-GAL4 > UAS.CD8::GFP	g
	VT061933-GAL4 > UAS.DenMark,Syt::GFP	h
	R22C06-LexA>LexAop.P2X2, VT061933-GAL4>UAS.GCamp6m	j
	R22C06-LexA/ +, VT061933-GAL4>UAS.GCamp6m	j
	VT061933-GAL4 > UAS.Kir2.1	k,l
	VT061933-GAL4/ +	k,l
	UAS.Kir2.1/ +	l
3	VT061933-GAL4 > UAS.Kir2.1	d,g
	UAS.Kir2.1/ +	e,g
	VT061933-GAL4/ +	e,g
	WT	h
	Orco^{2}	h
4	VT061933-GAL4 > UAS.CD8::GFP	b-e,g,h,k
	VT040053-GAL4.DBD; R22C06 AD> UAS.CD8::GFP	i,j
5	VT061933-GAL4/ +	b-e,g
	VT061933-GAL4 > UAS.Kir2.1	b-d,f,g
	UAS.Kir2.1/ +	c-e,g
7	VT019018 AD; VT017411-GAL4.DBD / +	e
	VT019018 AD; VT017411-GAL4.DBD > UAS.Kir2.1	e
	VT023490 AD; R38F04-GAL4.DBD > UAS.Kir2.1	e

Supplementary Table 4. Table of Genotypes
