# Supplementary material for: Rapid threat assessment in the Drosophila thermosensory system
Source: Nat Commun. 2023 Nov 3;14:7067. doi: 10.1038/s41467-023-42864-5 (PMC10624821; doi:10.1038/s41467-023-42864-5)
Supplement: Supplementary file 3 — Description of Additional Supplementary Files [file 41467_2023_42864_MOESM3_ESM.pdf]

**Description of Additional Supplementary Files**

File Name: Supplementary Data 1
Description: Statistical information regarding Figures 1-5, 7 and Supplementary Figure 3. Columns denote Figure, Figure panel, Statistical test used, Condition or genotype, Test Statistic, Sample size, P value, Degrees of freedom (where appropriate), Levene's Test (where appropriate), Kolmogorov–Smirnov normality test (where appropriate). For all behavioral experiments involving 2-ways ANOVA N = [x, x, x] refers to the Ns of experimental and control genotypes, in the order [Gal4/Kir, Gal4/+, Kir/+]. Condition** refers to the order of temperatures used in 2-choiche experiments, as in [25°C vs 30°C, 25°C vs 35°C, 25°C vs 40°C].
