# Supplementary material for: Rapid threat assessment in the Drosophila thermosensory system
Source: Nat Commun. 2023 Nov 3;14:7067. doi: 10.1038/s41467-023-42864-5 (PMC10624821; doi:10.1038/s41467-023-42864-5)
Supplement: Supplementary file 5 — Reporting Summary [file 41467_2023_42864_MOESM5_ESM.pdf]

nature portfolio

Corresponding author(s): Marco Gallio

Last updated by author(s): Aug 11, 2023

Reporting Summary

Nature Portfolio wishes to improve the reproducibility of the work that we publish. This form provides structure for consistency and transparency in reporting. For further information on Nature Portfolio policies, see our Editorial Policies and the Editorial Policy Checklist.

Statistics

For all statistical analyses, confirm that the following items are present in the figure legend, table legend, main text, or Methods section.

n/a	Confirmed	
☐	☒	The exact sample size (*n*) for each experimental group/condition, given as a discrete number and unit of measurement
☐	☒	A statement on whether measurements were taken from distinct samples or whether the same sample was measured repeatedly
☐	☒	The statistical test(s) used AND whether they are one- or two-sided<br>*Only common tests should be described solely by name; describe more complex techniques in the Methods section.*
☐	☒	A description of all covariates tested
☐	☒	A description of any assumptions or corrections, such as tests of normality and adjustment for multiple comparisons
☐	☒	A full description of the statistical parameters including central tendency (e.g. means) or other basic estimates (e.g. regression coefficient) AND variation (e.g. standard deviation) or associated estimates of uncertainty (e.g. confidence intervals)
☐	☒	For null hypothesis testing, the test statistic (e.g. *F*, *t*, *r*) with confidence intervals, effect sizes, degrees of freedom and *P* value noted<br>*Give P values as exact values whenever suitable.*
☒	☐	For Bayesian analysis, information on the choice of priors and Markov chain Monte Carlo settings
☐	☒	For hierarchical and complex designs, identification of the appropriate level for tests and full reporting of outcomes
☒	☐	Estimates of effect sizes (e.g. Cohen's *d*, Pearson's *r*), indicating how they were calculated

Our web collection on statistics for biologists contains articles on many of the points above.

Software and code

Policy information about availability of computer code

Data collection	Two-photon and confocal images were processed using Fiji. Light stimuli were triggered using a TTL pulse with pClamp software delivered to the LED driver. For electrophysiology experiments, images were acquired with an upright Zeiss Examiner.Z1 microscope with a Zeiss W Plan-Apochromat 40×0.9 numerical aperture water immersion objective at 512 pixels × 512 pixels resolution using PrairieView software v. 5.2 (Bruker). Current clamp recordings were acquired with Clampex software v.9.2.1.9 (Axon Instruments). For behavioral assays, the temperature of the Peltier was controlled using a custom MATLAB script and movements were recorded using a Chameleon3 USB camera (FLIR). To determine synaptic connectivity between TPN-IIIs, TLHONs, and LHPV2a1 etc., we used Python scripts to query Neuprint.
Data analysis	For navigation and olfactometry experiments, fly tracking and data analysis were performed using Python (v. 2.7), where the OpenCV package was used to do basic image processing. The data was then processed in MATLAB. Individual fly behavior analysis was done using Python scripts. Data from membrane potential recordings was analyzed off line using Axograph and standard scripts in Igor Pro and MATLAB. Action potentials (spikes) were detected standard scripts in Igor Pro using Neuromatic v2.6i plug-in. Custom code is available through the GallioLab GitHub (https://github.com/MarcoGallio/GallioLab).

For manuscripts utilizing custom algorithms or software that are central to the research but not yet described in published literature, software must be made available to editors and reviewers. We strongly encourage code deposition in a community repository (e.g. GitHub). See the Nature Portfolio guidelines for submitting code & software for further information.

Data

Policy information about availability of data

All manuscripts must include a data availability statement. This statement should provide the following information, where applicable:

- Accession codes, unique identifiers, or web links for publicly available datasets
- A description of any restrictions on data availability
- For clinical datasets or third party data, please ensure that the statement adheres to our policy

A data availability statement containing the required information was included in the manuscript. The data generated during the current study are available as a source data file and from the corresponding author on request. The following databases were used in this study: Flylight (https://www.janelia.org/project-team/flylight),Vienna Drosophila Resource Center (https://shop.vbc.ac.at/vdrc_store/) and neuPrint (https://neuprint.janelia.org/). Custom code is available through the GallioLab GitHub (https://github.com/MarcoGallio/GallioLab).

Research involving human participants, their data, or biological material

Policy information about studies with human participants or human data. See also policy information about sex, gender (identity/presentation), and sexual orientation and race, ethnicity and racism.

Reporting on sex and gender	N/A
Reporting on race, ethnicity, or other socially relevant groupings	N/A
Population characteristics	N/A
Recruitment	N/A
Ethics oversight	N/A

Note that full information on the approval of the study protocol must also be provided in the manuscript.

Field-specific reporting

Please select the one below that is the best fit for your research. If you are not sure, read the appropriate sections before making your selection.

☒ Life sciences ☐ Behavioural & social sciences ☐ Ecological, evolutionary & environmental sciences

For a reference copy of the document with all sections, see nature.com/documents/nr-reporting-summary-flat.pdf

Life sciences study design

All studies must disclose on these points even when the disclosure is negative.

Sample size	The sample size was chosen based on field standards from similar experiments in many previous studies
Data exclusions	No data were excluded from the analysis.
Replication	For the electrophysiological experiments, repeated measurements were taken from a number of cells and animals indicated in the corresponding Figure legend of panels. For all other experiments, results were replicated in different flies across datasets and were reproduced on a minimum of 5 animals/condition.
Randomization	No differential treatment was used so no standard randomization applies. Experimental genotypes and controls where identified using genetic markers as appropriate. Testing of controls and experimental groups was interspersed.
Blinding	The experimenters were not blind to genotypes. Blinding was not relevant since data analysis was automated eliminating potential operator bias.

Reporting for specific materials, systems and methods

We require information from authors about some types of materials, experimental systems and methods used in many studies. Here, indicate whether each material, system or method listed is relevant to your study. If you are not sure if a list item applies to your research, read the appropriate section before selecting a response.

Materials & experimental systems

n/a	Involved in the study	
☒	☐	Antibodies
☒	☐	Eukaryotic cell lines
☒	☐	Palaeontology and archaeology
☐	☒	Animals and other organisms
☒	☐	Clinical data
☒	☐	Dual use research of concern
☒	☐	Plants

Methods

n/a	Involved in the study	
☒	☐	ChIP-seq
☒	☐	Flow cytometry
☒	☐	MRI-based neuroimaging

Animals and other research organisms

Policy information about studies involving animals; ARRIVE guidelines recommended for reporting animal research, and Sex and Gender in Research

Laboratory animals	The Table of Genotypes (Suppementary material Table IV) provides a detailed description of the fly genotypes used in each experiment and origin of the transgenic stocks, including references to specific figure panels. Details on the generation of transgenic stocks created for this work are found in the corresponding Methods' section. Specific fly age is specified in methods.
Wild animals	No wild animals were used in this study
Reporting on sex	N/A
Field-collected samples	No field collected samples were used in this study
Ethics oversight	No ethic approval was required because Drosophila melanogaster was used as experimental animal.

Note that full information on the approval of the study protocol must also be provided in the manuscript.
